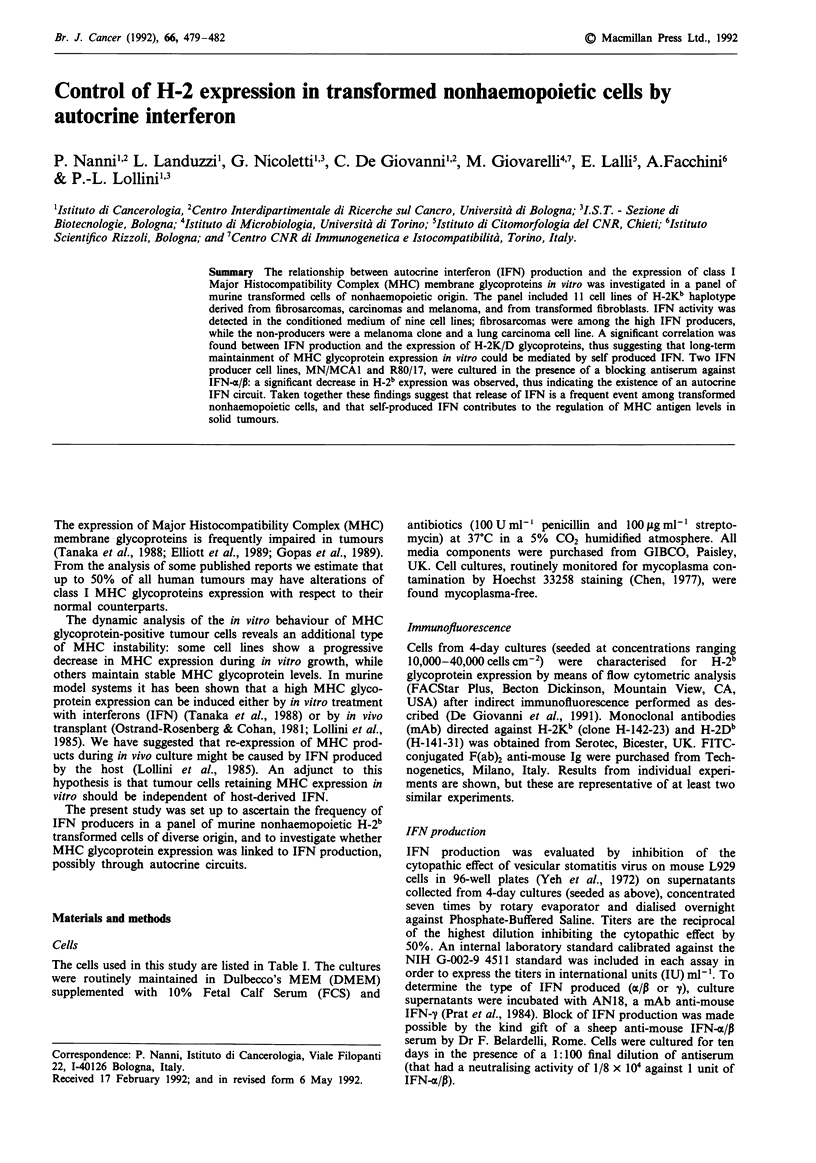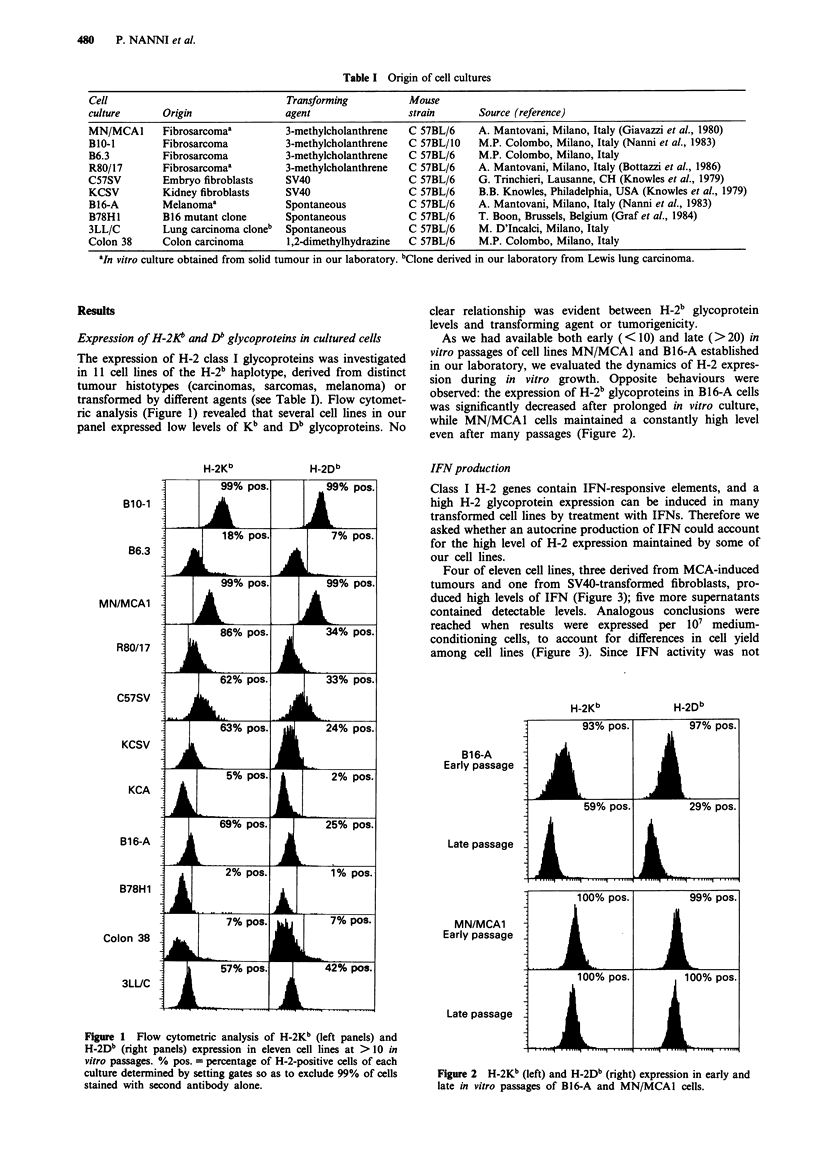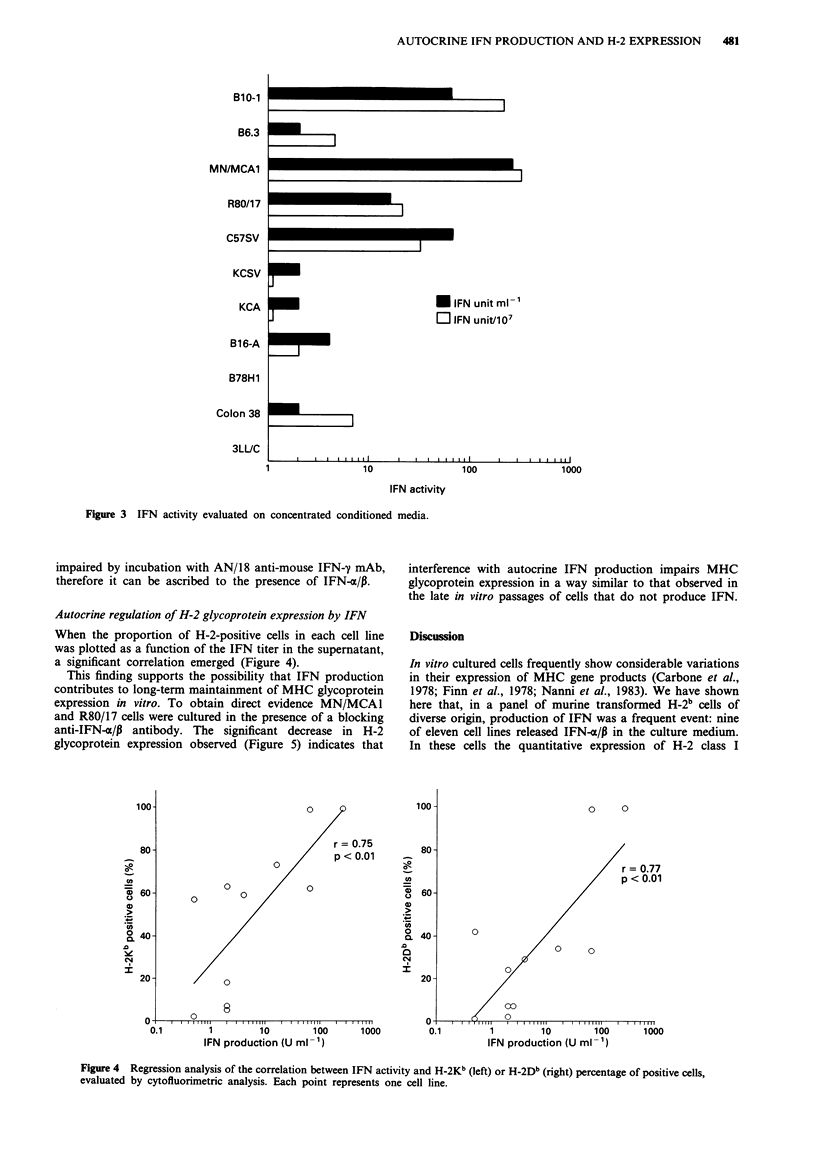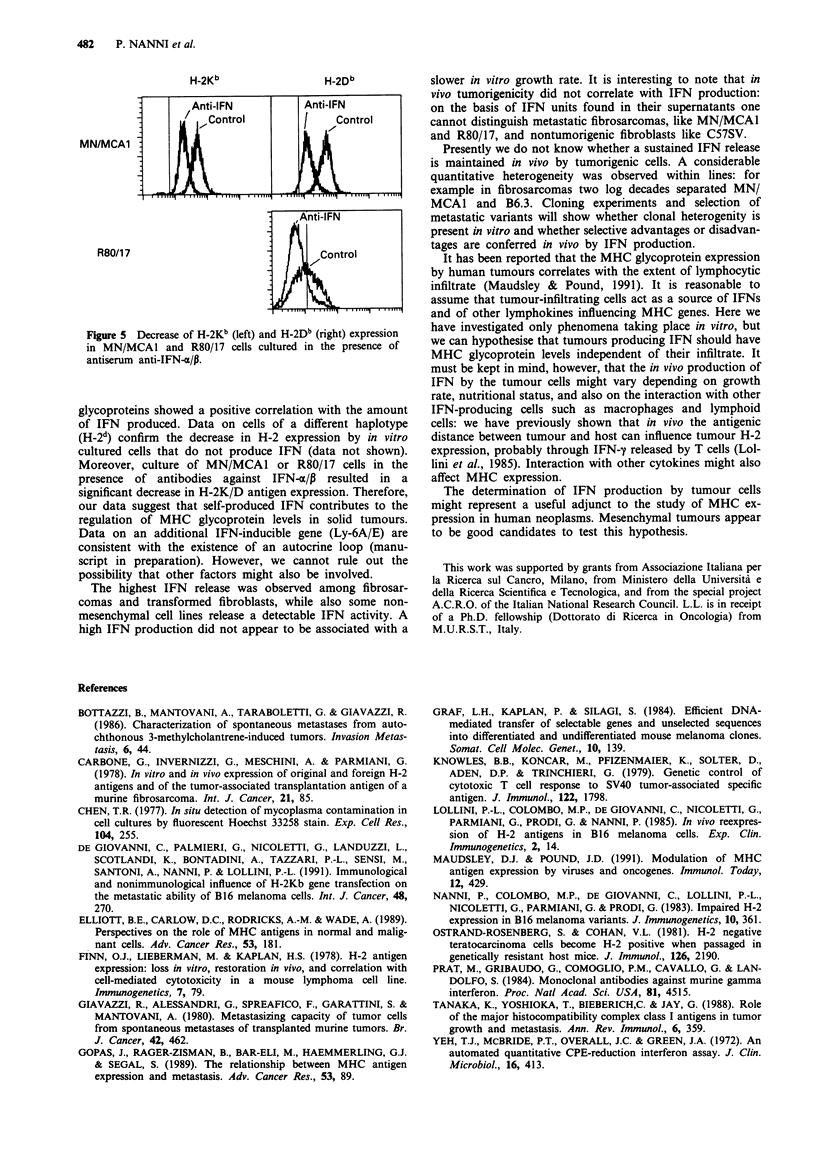# Control of H-2 expression in transformed nonhaemopoietic cells by autocrine interferon.

**DOI:** 10.1038/bjc.1992.299

**Published:** 1992-09

**Authors:** P. Nanni, L. Landuzzi, G. Nicoletti, C. De Giovanni, M. Giovarelli, E. Lalli, A. Facchini, P. L. Lollini

**Affiliations:** Istituto di Cancerologia, Università di Bologna, Italy.

## Abstract

The relationship between autocrine interferon (IFN) production and the expression of class I Major Histocompatibility Complex (MHC) membrane glycoproteins in vitro was investigated in a panel of murine transformed cells of nonhaemopoietic origin. The panel included 11 cell lines of H-2Kb haplotype derived from fibrosarcomas, carcinomas and melanoma, and from transformed fibroblasts. IFN activity was detected in the conditioned medium of nine cell lines; fibrosarcomas were among the high IFN producers, while the non-producers were a melanoma clone and a lung carcinoma cell line. A significant correlation was found between IFN production and the expression of H-2K/D glycoproteins, thus suggesting that long-term maintainment of MHC glycoprotein expression in vitro could be mediated by self produced IFN. Two IFN producer cell lines, MN/MCA1 and R80/17, were cultured in the presence of a blocking antiserum against IFN-alpha/beta: a significant decrease in H-2b expression was observed, thus indicating the existence of an autocrine IFN circuit. Taken together these findings suggest that release of IFN is a frequent event among transformed nonhaemopoietic cells, and that self-produced IFN contributes to the regulation of MHC antigen levels in solid tumours.


Br. J. Cancer (1992), 66, 479-482 © Macmillan Press Ltd., 1992

### Control of H-2 expression in transformed nonhaemopoietic cells by autocrine interferon

P. Nanni[1,2] L. Landuzzi[1], G. Nicoletti[1,3], C. De Giovanni[1,2], M. Giovarelli[4,7], E. Lalli[5], A.Facchini[6] & P.-L. Lollini[1,3]

[1]Istituto di Cancerologia, [2]Centro Interdipartimentale di Ricerche sul Cancro, Università di Bologna; [3]I.S.T. - Sezione di Biotecnologie, Bologna; [4]Istituto di Microbiologia, Università di Torino; [5]Istituto di Citomorfologia del CNR, Chieti; [6]Istituto Scientifico Rizzoli, Bologna; and [7]Centro CNR di Immunogenetica e Istocompatibilità, Torino, Italy.

**Summary** The relationship between autocrine interferon (IFN) production and the expression of class I Major Histocompatibility Complex (MHC) membrane glycoproteins *in vitro* was investigated in a panel of murine transformed cells of nonhaemopoietic origin. The panel included 11 cell lines of H-2K$^b$ haplotype derived from fibrosarcomas, carcinomas and melanoma, and from transformed fibroblasts. IFN activity was detected in the conditioned medium of nine cell lines; fibrosarcomas were among the high IFN producers, while the non-producers were a melanoma clone and a lung carcinoma cell line. A significant correlation was found between IFN production and the expression of H-2K/D glycoproteins, thus suggesting that long-term maintainment of MHC glycoprotein expression *in vitro* could be mediated by self produced IFN. Two IFN producer cell lines, MN/MCA1 and R80/17, were cultured in the presence of a blocking antiserum against IFN-$\alpha/\beta$: a significant decrease in H-2$^b$ expression was observed, thus indicating the existence of an autocrine IFN circuit. Taken together these findings suggest that release of IFN is a frequent event among transformed nonhaemopoietic cells, and that self-produced IFN contributes to the regulation of MHC antigen levels in solid tumours.

The expression of Major Histocompatibility Complex (MHC) membrane glycoproteins is frequently impaired in tumours (Tanaka *et al.*, 1988; Elliott *et al.*, 1989; Gopas *et al.*, 1989). From the analysis of some published reports we estimate that up to 50% of all human tumours may have alterations of class I MHC glycoproteins expression with respect to their normal counterparts.

The dynamic analysis of the *in vitro* behaviour of MHC glycoprotein-positive tumour cells reveals an additional type of MHC instability: some cell lines show a progressive decrease in MHC expression during *in vitro* growth, while others maintain stable MHC glycoprotein levels. In murine model systems it has been shown that a high MHC glycoprotein expression can be induced either by *in vitro* treatment with interferons (IFN) (Tanaka *et al.*, 1988) or by *in vivo* transplant (Ostrand-Rosenberg & Cohan, 1981; Lollini *et al.*, 1985). We have suggested that re-expression of MHC products during *in vivo* culture might be caused by IFN produced by the host (Lollini *et al.*, 1985). An adjunct to this hypothesis is that tumour cells retaining MHC expression *in vitro* should be independent of host-derived IFN.

The present study was set up to ascertain the frequency of IFN producers in a panel of murine nonhaemopoietic H-2$^b$ transformed cells of diverse origin, and to investigate whether MHC glycoprotein expression was linked to IFN production, possibly through autocrine circuits.

### Materials and methods

### Cells

The cells used in this study are listed in Table I. The cultures were routinely maintained in Dulbecco's MEM (DMEM) supplemented with 10% Fetal Calf Serum (FCS) and antibiotics (100 U ml$^{-1}$ penicillin and 100 $\mu$g ml$^{-1}$ streptomycin) at 37°C in a 5% $CO_2$ humidified atmosphere. All media components were purchased from GIBCO, Paisley, UK. Cell cultures, routinely monitored for mycoplasma contamination by Hoechst 33258 staining (Chen, 1977), were found mycoplasma-free.

### Immunofluorescence

Cells from 4-day cultures (seeded at concentrations ranging 10,000-40,000 cells cm$^{-2}$) were characterised for H-2$^b$ glycoprotein expression by means of flow cytometric analysis (FACStar Plus, Becton Dickinson, Mountain View, CA, USA) after indirect immunofluorescence performed as described (De Giovanni *et al.*, 1991). Monoclonal antibodies (mAb) directed against H-2K$^b$ (clone H-142-23) and H-2D$^b$ (H-141-31) was obtained from Serotec, Bicester, UK. FITC-conjugated F(ab)$_2$ anti-mouse Ig were purchased from Technogenetics, Milano, Italy. Results from individual experiments are shown, but these are representative of at least two similar experiments.

### IFN production

IFN production was evaluated by inhibition of the cytopathic effect of vesicular stomatitis virus on mouse L929 cells in 96-well plates (Yeh *et al.*, 1972) on supernatants collected from 4-day cultures (seeded as above), concentrated seven times by rotary evaporator and dialised overnight against Phosphate-Buffered Saline. Titers are the reciprocal of the highest dilution inhibiting the cytopathic effect by 50%. An internal laboratory standard calibrated against the NIH G-002-9 4511 standard was included in each assay in order to express the titers in international units (IU) ml$^{-1}$. To determine the type of IFN produced ($\alpha/\beta$ or $\gamma$), culture supernatants were incubated with AN18, a mAb anti-mouse IFN-$\gamma$ (Prat *et al.*, 1984). Block of IFN production was made possible by the kind gift of a sheep anti-mouse IFN-$\alpha/\beta$ serum by Dr F. Belardelli, Rome. Cells were cultured for ten days in the presence of a 1:100 final dilution of antiserum (that had a neutralising activity of 1/8 × 10$^4$ against 1 unit of IFN-$\alpha/\beta$).

Correspondence: P. Nanni, Istituto di Cancerologia, Viale Filopanti 22, I-40126 Bologna, Italy.
Received 17 February 1992; and in revised form 6 May 1992.

**Table I** Origin of cell cultures

| *Cell culture* | *Origin* | *Transforming agent* | *Mouse strain* | *Source (reference)* |
|---|---|---|---|---|
| MN/MCA1 | Fibrosarcoma[a] | 3-methylcholanthrene | C 57BL/6 | A. Mantovani, Milano, Italy (Giavazzi *et al.*, 1980) |
| B10-1 | Fibrosarcoma | 3-methylcholanthrene | C 57BL/10 | M.P. Colombo, Milano, Italy (Nanni *et al.*, 1983) |
| B6.3 | Fibrosarcoma | 3-methylcholanthrene | C 57BL/6 | M.P. Colombo, Milano, Italy |
| R80/17 | Fibrosarcoma[a] | 3-methylcholanthrene | C 57BL/6 | A. Mantovani, Milano, Italy (Bottazzi *et al.*, 1986) |
| C57SV | Embryo fibroblasts | SV40 | C 57BL/6 | G. Trinchieri, Lausanne, CH (Knowles *et al.*, 1979) |
| KCSV | Kidney fibroblasts | SV40 | C 57BL/6 | B.B. Knowles, Philadelphia, USA (Knowles *et al.*, 1979) |
| B16-A | Melanoma[a] | Spontaneous | C 57BL/6 | A. Mantovani, Milano, Italy (Nanni *et al.*, 1983) |
| B78H1 | B16 mutant clone | Spontaneous | C 57BL/6 | T. Boon, Brussels, Belgium (Graf *et al.*, 1984) |
| 3LL/C | Lung carcinoma clone[b] | Spontaneous | C 57BL/6 | M. D'Incalci, Milano, Italy |
| Colon 38 | Colon carcinoma | 1,2-dimethylhydrazine | C 57BL/6 | M.P. Colombo, Milano, Italy |

[a]*In vitro* culture obtained from solid tumour in our laboratory. [b]Clone derived in our laboratory from Lewis lung carcinoma.

### Results

### *Expression of H-2K$^b$ and D$^b$ glycoproteins in cultured cells*

The expression of H-2 class I glycoproteins was investigated in 11 cell lines of the H-2$^b$ haplotype, derived from distinct tumour histotypes (carcinomas, sarcomas, melanoma) or transformed by different agents (see Table I). Flow cytometric analysis (Figure 1) revealed that several cell lines in our panel expressed low levels of K$^b$ and D$^b$ glycoproteins. No clear relationship was evident between H-2$^b$ glycoprotein levels and transforming agent or tumorigenicity.

As we had available both early (<10) and late (>20) *in vitro* passages of cell lines MN/MCA1 and B16-A established in our laboratory, we evaluated the dynamics of H-2 expression during *in vitro* growth. Opposite behaviours were observed: the expression of H-2$^b$ glycoproteins in B16-A cells was significantly decreased after prolonged *in vitro* culture, while MN/MCA1 cells maintained a constantly high level even after many passages (Figure 2).

**Figure 1** Flow cytometric analysis of H-2K$^b$ (left panels) and H-2D$^b$ (right panels) expression in eleven cell lines at >10 *in vitro* passages. % pos. = percentage of H-2-positive cells of each culture determined by setting gates so as to exclude 99% of cells stained with second antibody alone.

### *IFN production*

Class I H-2 genes contain IFN-responsive elements, and a high H-2 glycoprotein expression can be induced in many transformed cell lines by treatment with IFNs. Therefore we asked whether an autocrine production of IFN could account for the high level of H-2 expression maintained by some of our cell lines.

Four of eleven cell lines, three derived from MCA-induced tumours and one from SV40-transformed fibroblasts, produced high levels of IFN (Figure 3); five more supernatants contained detectable levels. Analogous conclusions were reached when results were expressed per $10^7$ medium-conditioning cells, to account for differences in cell yield among cell lines (Figure 3). Since IFN activity was not

**Figure 2** H-2K$^b$ (left) and H-2D$^b$ (right) expression in early and late *in vitro* passages of B16-A and MN/MCA1 cells.

**Figure 3** IFN activity evaluated on concentrated conditioned media.

impaired by incubation with AN/18 anti-mouse IFN-γ mAb, therefore it can be ascribed to the presence of IFN-α/β.

*Autocrine regulation of H-2 glycoprotein expression by IFN*

When the proportion of H-2-positive cells in each cell line was plotted as a function of the IFN titer in the supernatant, a significant correlation emerged (Figure 4).

This finding supports the possibility that IFN production contributes to long-term maintainment of MHC glycoprotein expression *in vitro*. To obtain direct evidence MN/MCA1 and R80/17 cells were cultured in the presence of a blocking anti-IFN-α/β antibody. The significant decrease in H-2 glycoprotein expression observed (Figure 5) indicates that interference with autocrine IFN production impairs MHC glycoprotein expression in a way similar to that observed in the late *in vitro* passages of cells that do not produce IFN.

### Discussion

*In vitro* cultured cells frequently show considerable variations in their expression of MHC gene products (Carbone *et al.*, 1978; Finn *et al.*, 1978; Nanni *et al.*, 1983). We have shown here that, in a panel of murine transformed H-2$^b$ cells of diverse origin, production of IFN was a frequent event: nine of eleven cell lines released IFN-α/β in the culture medium. In these cells the quantitative expression of H-2 class I

**Figure 4** Regression analysis of the correlation between IFN activity and H-2K$^b$ (left) or H-2D$^b$ (right) percentage of positive cells, evaluated by cytofluorimetric analysis. Each point represents one cell line.

**Figure 5** Decrease of H-2K$^b$ (left) and H-2D$^b$ (right) expression in MN/MCA1 and R80/17 cells cultured in the presence of antiserum anti-IFN-α/β.

glycoproteins showed a positive correlation with the amount of IFN produced. Data on cells of a different haplotype (H-2$^d$) confirm the decrease in H-2 expression by *in vitro* cultured cells that do not produce IFN (data not shown). Moreover, culture of MN/MCA1 or R80/17 cells in the presence of antibodies against IFN-α/β resulted in a significant decrease in H-2K/D antigen expression. Therefore, our data suggest that self-produced IFN contributes to the regulation of MHC glycoprotein levels in solid tumours. Data on an additional IFN-inducible gene (Ly-6A/E) are consistent with the existence of an autocrine loop (manuscript in preparation). However, we cannot rule out the possibility that other factors might also be involved.

The highest IFN release was observed among fibrosarcomas and transformed fibroblasts, while also some non-mesenchymal cell lines release a detectable IFN activity. A high IFN production did not appear to be associated with a slower *in vitro* growth rate. It is interesting to note that *in vivo* tumorigenicity did not correlate with IFN production: on the basis of IFN units found in their supernatants one cannot distinguish metastatic fibrosarcomas, like MN/MCA1 and R80/17, and nontumorigenic fibroblasts like C57SV.

Presently we do not know whether a sustained IFN release is maintained *in vivo* by tumorigenic cells. A considerable quantitative heterogeneity was observed within lines: for example in fibrosarcomas two log decades separated MN/MCA1 and B6.3. Cloning experiments and selection of metastatic variants will show whether clonal heterogenity is present *in vitro* and whether selective advantages or disadvantages are conferred *in vivo* by IFN production.

It has been reported that the MHC glycoprotein expression by human tumours correlates with the extent of lymphocytic infiltrate (Maudsley & Pound, 1991). It is reasonable to assume that tumour-infiltrating cells act as a source of IFNs and of other lymphokines influencing MHC genes. Here we have investigated only phenomena taking place *in vitro*, but we can hypothesise that tumours producing IFN should have MHC glycoprotein levels independent of their infiltrate. It must be kept in mind, however, that the *in vivo* production of IFN by the tumour cells might vary depending on growth rate, nutritional status, and also on the interaction with other IFN-producing cells such as macrophages and lymphoid cells: we have previously shown that *in vivo* the antigenic distance between tumour and host can influence tumour H-2 expression, probably through IFN-γ released by T cells (Lollini *et al.*, 1985). Interaction with other cytokines might also affect MHC expression.

The determination of IFN production by tumour cells might represent a useful adjunct to the study of MHC expression in human neoplasms. Mesenchymal tumours appear to be good candidates to test this hypothesis.

This work was supported by grants from Associazione Italiana per la Ricerca sul Cancro, Milano, from Ministero della Università e della Ricerca Scientifica e Tecnologica, and from the special project A.C.R.O. of the Italian National Research Council. L.L. is in receipt of a Ph.D. fellowship (Dottorato di Ricerca in Oncologia) from M.U.R.S.T., Italy.

### References

BOTTAZZI, B., MANTOVANI, A., TARABOLETTI, G. & GIAVAZZI, R. (1986). Characterization of spontaneous metastases from autochthonous 3-methylcholantrene-induced tumors. *Invasion Metastasis*, **6**, 44.

CARBONE, G., INVERNIZZI, G., MESCHINI, A. & PARMIANI, G. (1978). *In vitro* and *in vivo* expression of original and foreign H-2 antigens and of the tumor-associated transplantation antigen of a murine fibrosarcoma. *Int. J. Cancer*, **21**, 85.

CHEN, T.R. (1977). *In situ* detection of mycoplasma contamination in cell cultures by fluorescent Hoechst 33258 stain. *Exp. Cell Res.*, **104**, 255.

DE GIOVANNI, C., PALMIERI, G., NICOLETTI, G., LANDUZZI, L., SCOTLANDI, K., BONTADINI, A., TAZZARI, P.-L., SENSI, M., SANTONI, A., NANNI, P. & LOLLINI, P.-L. (1991). Immunological and nonimmunological influence of H-2Kb gene transfection on the metastatic ability of B16 melanoma cells. *Int. J. Cancer*, **48**, 270.

ELLIOTT, B.E., CARLOW, D.C., RODRICKS, A.-M. & WADE, A. (1989). Perspectives on the role of MHC antigens in normal and malignant cells. *Adv. Cancer Res.*, **53**, 181.

FINN, O.J., LIEBERMAN, M. & KAPLAN, H.S. (1978). H-2 antigen expression: loss *in vitro*, restoration *in vivo*, and correlation with cell-mediated cytotoxicity in a mouse lymphoma cell line. *Immunogenetics*, **7**, 79.

GIAVAZZI, R., ALESSANDRI, G., SPREAFICO, F., GARATTINI, S. & MANTOVANI, A. (1980). Metastasizing capacity of tumor cells from spontaneous metastases of transplanted murine tumors. *Br. J. Cancer*, **42**, 462.

GOPAS, J., RAGER-ZISMAN, B., BAR-ELI, M., HAEMMERLING, G.J. & SEGAL, S. (1989). The relationship between MHC antigen expression and metastasis. *Adv. Cancer Res.*, **53**, 89.

GRAF, L.H., KAPLAN, P. & SILAGI, S. (1984). Efficient DNA-mediated transfer of selectable genes and unselected sequences into differentiated and undifferentiated mouse melanoma clones. *Somat. Cell Molec. Genet.*, **10**, 139.

KNOWLES, B.B., KONCAR, M., PFIZENMAIER, K., SOLTER, D., ADEN, D.P. & TRINCHIERI, G. (1979). Genetic control of cytotoxic T cell response to SV40 tumor-associated specific antigen. *J. Immunol.*, **122**, 1798.

LOLLINI, P.-L., COLOMBO, M.P., DE GIOVANNI, C., NICOLETTI, G., PARMIANI, G., PRODI, G. & NANNI, P. (1985). *In vivo* reexpression of H-2 antigens in B16 melanoma cells. *Exp. Clin. Immunogenetics*, **2**, 14.

MAUDSLEY, D.J. & POUND, J.D. (1991). Modulation of MHC antigen expression by viruses and oncogenes. *Immunol. Today*, **12**, 429.

NANNI, P., COLOMBO, M.P., DE GIOVANNI, C., LOLLINI, P.-L., NICOLETTI, G., PARMIANI, G. & PRODI, G. (1983). Impaired H-2 expression in B16 melanoma variants. *J. Immunogenetics*, **10**, 361.

OSTRAND-ROSENBERG, S. & COHAN, V.L. (1981). H-2 negative teratocarcinoma cells become H-2 positive when passaged in genetically resistant host mice. *J. Immunol.*, **126**, 2190.

PRAT, M., GRIBAUDO, G., COMOGLIO, P.M., CAVALLO, G. & LANDOLFO, S. (1984). Monoclonal antibodies against murine gamma interferon. *Proc. Natl Acad. Sci. USA*, **81**, 4515.

TANAKA, K., YOSHIOKA, T., BIEBERICH,C. & JAY, G. (1988). Role of the major histocompatibility complex class I antigens in tumor growth and metastasis. *Ann. Rev. Immunol.*, **6**, 359.

YEH, T.J., MCBRIDE, P.T., OVERALL, J.C. & GREEN, J.A. (1972). An automated quantitative CPE-reduction interferon assay. *J. Clin. Microbiol.*, **16**, 413.